# Kyste osseux essentiel du calcanéum découvert à l'occasion d’une entorse de la cheville: risque et prise en charge

**DOI:** 10.11604/pamj.2017.27.113.12912

**Published:** 2017-06-14

**Authors:** Mustafa Nkaoui, Amine El Yazidi

**Affiliations:** 1 Service de Chirurgie Orthopédique et de Traumatologie, CHU Ibn Sina, Université Mohammed V Souissi, Rabat, Maroc; 2 Service de Chirurgie, Orthopédique et de Traumatologie, Centre Hospitalier de Beauvais, France

**Keywords:** Kyste osseux essentiel, calcanéum, curetage, Essential bone cyst, calcaneus, curettage

## Image en médecine

Nous rapportons le cas d'une jeune fille âgée de 16 ans, reçue aux urgences pour un traumatisme de sa cheville gauche lors d'un accident de sport. Le diagnostic d'une entorse simple a été évoqué cliniquement. La radiologie standard a objectivé une lésion ostéolytique lacunaire au niveau du calcanéum sans visualisation de trait de fracture (Panel A). L'IRM du pied a confirmé le caractère kystique de la lésion avec une composante hémorragique (Panel B). La prise en charge en différé a consisté en un curetage comblement à l'aide de substitut osseux de synthèse (Panel C, D, E, F) avec pose d'un plâtre pendant 3 semaines sans appui suivie d'une rééducation fonctionnelle de la cheville. L'étude histologique était caractéristique de kyste essentiel du calcanéum. Le résultat était satisfaisant avec une bonne consolidation sans récidive et sans complications et reprise du sport au 3^ème^ mois. Le kyste osseux essentiel du calcanéum est une lésion rare et bénigne, souvent asymptomatique et de découverte fortuite. Le principal risque évolutif étant la fracture pathologique justifiant un traitement chirurgical à la fois curatif et préventif.

**Figure 1:**
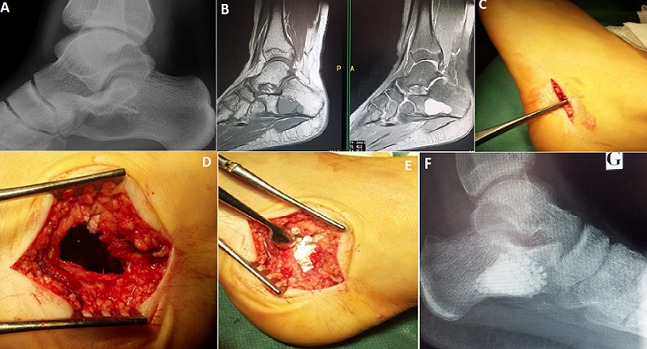
(A) radiographie standard du pied gauche montrant une image lacunaire ostéolytique du calcanéum comportant de fines cloisons avec corticale amincie; (B) IRM du pied gauche: formation liquidienne polylobée bien limitée interessant les tiers antérieur et moyen du calcanéum en hyposignal modéré en T1 et hypersignal liquidien en T2 associé à un niveau liquidien postérieur faisant suspecter une composante hémorragique; (C) image péropératoire: répérage scopique de la voie d’abord; (D) image péropératoire: trépanation de la face externe de l’os et curetage évidement de la cavité; (E) image péropératoire: comblement de la cavité par une greffe à partir de subtituts osseux; (F) contrôle radiologique post opératoire à un mois

